# Virus discovery in sandflies, from field studies to phylogenetic tree building: classic methods versus novel methods, what do we need to address public health impact?

**DOI:** 10.1186/1756-3305-7-S1-O10

**Published:** 2014-04-01

**Authors:** L Bichaud, C Alkan, G Piorkowski, E Zhioua, I Bitam, A Izri, V Moin Vaziri, B Alten, Y Ozbel, OE Kasap, X De Lamballerie, RN Charrel

**Affiliations:** 1 Aix Marseille Universite, IRD French Institute of Research for Development, EHESP French School of Public Health, EPV UMR_D 190 "Emergence des Pathologies Virales", 13385, Marseille, France; 2 Institut Pasteur, Tunis, Tunisia; 3 Institut Pasteur, Algiers, Algeria; 4 Shahid Beheshti University of Medical Sciences, Tehran, Iran; 5 Hacettepe University, Ankara, Turkey; 6 Ege University, Izmir, Turkey

## 

Phlebotomine sandflies are vectors of a large variety of arboviruses, mostly in the genus *Phlebovirus*. Many were discovered during the last decade, but were only partially sequenced because of extremely high genetic diversity. Next-Generation sequencing (NGS) is a key tool to achieve rapid sequencing of these viruses. Here we present the results obtained with a custom-made automated platform for high throughput processing (HTP) of field-trapped sandflies.

The aim is to present an integrated approach to discover new/existing viruses vectored by sandflies, to characterize these viruses by complete genome sequencing, and to study their potential public health impact for humans and non human vertebrates.

Sandflies were collected in France, Tunisia, Algeria, Turkey and Iran. They were processed on a custom-made automated HTP platform. RT-PCR assays targeting conserved regions within the genus *Phlebovirus *were performed both directly on sandfly extracted RNA and on supernatant of Vero cells, and further sequenced. Sandflies and cell culture that were PCR-positive, as well as supernatant of flasks with cytopathic effect were sequenced using a *de novo *protocol onto a Ion Torrent PGM sequencer.

A total of 29,664 sandflies were collected and processed onto the platform. Origin and number of specimens are as follows: France (n = 3,087), Tunisia (n = 8,206), Algeria (n = 1,283), Turkey (n = 12,000), Iran (n = 5,089). We isolated 9 virus strains in France (2 Toscana, 7 Massilia), 10 strains in Tunisia (2 Toscana, 5 Punique, 3 new viruses still unnamed), 1 strain in Algeria (1 Toscana), 2 strains in Turkey (new phlebovirus, provisionally named Damyeri), 1 strain in Iran (new phlebovirus, provisionally named Dashli). Analysis using complete gene sequences allowed to produce full protein phylograms for isolated virus strains. Seroprevalence studies were performed to define whether these viruses infect humans and non human vertebrates in regions where sandflies were trapped or not. Seroprevalence results will be presented for France and Tunisia.

A total of 23 strains were isolated, 6 of which correspond to new sandfly-borne phleboviruses that have been completely sequenced through NGS. Screening field-trapped sandflies is suitable to inventory recognized and new viruses. Combining cell culture for isolation and NGS for sequencing is cost-effective and adapted to process high quantities of insects. Serological tools are a rapid and easy method to address potential public health impact which must be confirmed by molecular diagnostic derived from complete sequence data obtained through NGS.

